# A Maxim for the Dentist

**Published:** 1854-01

**Authors:** John Harris

**Affiliations:** Salem, O.


*A Maxim for the Dentist.* By Dr. John Harris, Salem, O.

It is true of all professions, as well as all other kınds of business, but most especially is it true in the practice of dentistry, that "*whatever is worth doing at all is worth doing well.*"* Could I impress this maxim in the full extent of its meaning and import upon the minds of all the members of the dental profession, I would consider it one of the grandest achievements possible toward the ultimate success of our business as a profession.

By introducing the above maxim into all his operations, the dentist would be most amply compensated by the increased amount of business which such a course would almost necessarily procure, because every well-performed operation would be the means of procuring at least half a dozen new cases, which in the event of his failure in the first, would have gone elsewhere, or neglected to have the operations performed at all.

The adoption of our maxim would be of greater moment still to the community than to the operator- It makes a vast difference to a patient whether he, after submitting to perhaps a painful operation, and paying his money for it, is really injured in point of health and comfort, as well as purse, or whether the services received from the dentist are worth ten times what they cost.

To dental surgery as a profession, it would be of inestimable value. Its direct tendency would be to elevate and to extend the sphere of its usefulness; and it would augment, to a surprising degree, the demand for our services, for it is well known to every dentist that less than one-half of the work really needed in almost all sections of the country is attended to at all, owing, in a great degree, to the manner in which much of the work is done.

To the truly high-miuded, conscientious man, the adoption of our maxim would afford a reward still greater, and of a character superior to any yet mentioned, namely, the consciousness of doing his duty, and being really useful to

* The above maxim is wotrh setting in *gold.*—Ed.

others. "*Whatever is worth doing at all is worth doing well.*"

Let me illustrate. Suppose we are called upon to fill the cavity in a carious tooth. Can it be *well* done? is frequently the first question of our patient, and undoubtedly should be one of the first questions in the mind of the operator. Our rule should be—unless it can be well done, not to do it at all. Suppose it to be a cavity in the lateral surface of an incisor tooth, difficult to approach, and much larger at the orifice than the interior of the cavity, the tooth sensitive, our patient nervous, fidgety, and *im*patient, the day cloudy, and he from a distance. After we have bent over our case some two hours, excavating, or *trying* to excavate, in order to secure a proper shape to retain the filling, our patient's *patience*, we perceive, is beginning to flag, and we begin to wish we had hold of an axe, a plough, a grubbing hoe, or anything but an excavator, the question begins to present itself—Will it do? Can I make it any better? We will suppose that we concluded to make it do, just before we had the cavity quite properly prepared. With much difficulty we introduce our gold, entertaining some pretty well grounded fears that our case would not terminate as we should wish, yet hoping that the job will prove to be well done. Of one thing we feel certain—we labored hard at it. In the course of a few weeks, our patient, in carelessly using his penknife for a toothpick, displaces the filling, and when inquired of by his neighbor how he liked Mr. —— as a dentist, he replies that "he can not be good at his business; for he worked a long time, plugging a little tooth in my head, for which I paid him a dollar, and it came out in a short time." The neighbor replies—"My wife needs a set of teeth, but I guess I must go somewhere else; if Mr. —— can't plug a tooth, of course he cannot make a good whole set."

On the other hand, suppose we had persevered, and filled the tooth so that we knew it was *well* done, we might have been spared those unpleasant misgivings which annoyed us every time we thought of the case, we would have secured the neighbor's job of a whole set, and been spared the vexatious task of refilling the same tooth gratuitously.

The more experience I have, the more I am impressed with the importance of dentists doing their work well. Many of us, I have no doubt, can look back upon operations which proved to be deficient, and where we ought to have done better; but who among us ever regretted having performed an operation too well?

My attention was called to this subject by observing the manner in which a cavity was filled, in a tooth which I lately had occasion to extract, on account of periosteal inflammation. The cavity had been so well excavated, and the gold so densely compacted, that it seemed as if the filling might have remained in for a life-time, had the tooth been otherwise healthy. I know not who the operator was, but I have very little doubt of his success, if he performs all his operations as well as he did this.

My advice to students and to dentists everywhere would be, not to rest satisfied short of a thorough acquaintance with the principles, as well as the best mode of practice known to the profession. The idea too prevalent, even at this day, of picking up dentistry, to make some money with, is most ruinous upon the profession, as well as the community. It is almost sure to result in the disgrace and injury of the perpetrator, and it destroys that confidence, in the public mind, which is indispensable to success.

